# Melatonin Stimulates Dendrite Formation and Complexity in the Hilar Zone of the Rat Hippocampus: Participation of the Ca^++^/Calmodulin Complex

**DOI:** 10.3390/ijms16011907

**Published:** 2015-01-16

**Authors:** Aline Domínguez-Alonso, Marcela Valdés-Tovar, Héctor Solís-Chagoyán, Gloria Benítez-King

**Affiliations:** 1 Laboratorio de Neurofarmacología, Subdirección de Investigaciones Clínicas, Instituto Nacional de Psiquiatría Ramón de la Fuente Muñiz, Calzada México-Xochimilco No. 101, Col. San Lorenzo-Huipulco, CP 14370 Tlalpan, DF, Mexico; E-Mails: aline.dmgzalonso@gmail.com (A.D.-A.); mvaldes@imp.edu.mx (M.V.-T.); hecsolch@imp.edu.mx (H.S.-C.); 2 Posgrado en Ciencias Biológicas, Universidad Nacional Autónoma de México, Av. Ciudad Universitaria 3000, CP 04360 Coyoacán, DF, Mexico

**Keywords:** melatonin, dendrites, calmodulin-kinase II, calmodulin, hippocampus, neuropsychiatric disorders

## Abstract

Melatonin (MEL), the main product synthesized by the pineal gland, stimulates early and late stages of neurodevelopment in the adult brain. MEL increases dendrite length, thickness and complexity in the hilar and mossy neurons of hippocampus. Dendrite formation involves activation of Ca^2+^/Calmodulin (CaM)-dependent kinase II (CaMKII) by CaM. Previous work showed that MEL increased the synthesis and translocation of CaM, suggesting that MEL activates CaM-dependent enzymes by this pathway. In this work we investigated whether MEL stimulates dendrite formation by CaMKII activation in organotypic cultures from adult rat hippocampus. We found that the CaMKII inhibitor, KN-62, abolished the MEL stimulatory effects on dendritogenesis and that MEL increased the relative amount of CaM in the soluble fraction of hippocampal slices. Also, PKC inhibition abolished dendritogenesis, while luzindole, an antagonist of MEL receptors (MT1/2), partially blocked the effects of MEL. Moreover, autophosphorylation of CaMKII and PKC was increased in presence of MEL, as well as phosphorylation of ERK1/2. Our results indicate that MEL stimulates dendrite formation through CaMKII and the translocation of CaM to the soluble fraction. Dendritogenesis elicited by MEL also required PKC activation, and signaling through MT1/2 receptors was partially involved. Data strongly suggest that MEL could repair the loss of hippocampal dendrites that occur in neuropsychiatric disorders by increasing CaM levels and activation of CaMKII.

## 1. Introduction

One common feature in several neuropsychiatric disorders is that they present brain structural alterations. In particular, a decreased hippocampal volume has been described in major depression, schizophrenia, and Alzheimer’s disease [1,2,3,4,5]. Moreover, a loss of synaptic connectivity between the prefrontal cortex and the hippocampus has been described in these diseases [6,7,8,9]. Therefore, in recent years, research on new therapeutic alternatives for the treatment of neuropsychiatric disorders has been focused on molecules, hormones and differentiation factors capable of eliciting neurodevelopment in the adult brain, in an attempt to repair the damaged circuitry [10,11,12]. Among these compounds, melatonin (*N*-acetyl-5-methoxytryptamine; MEL) has been found to stimulate neurogenesis and neuronal survival in the dentate gyrus of the hippocampus [13,14,15,16]. In addition, this indoleamine prompts dendrite formation and maturation in new formed neurons and in preexisting adult interneurons in the hilar zone of the hippocampal formation in organotypic culture models as well as in injured brain tissue [17,18].

Dendrite formation is a complex process in which cytoskeletal rearrangements take place in response to kinase activation downstream membrane receptors or by transient Ca^2+^ signaling [19,20,21]. In particular, Ca^2+^/CaM kinase II (CaMKII) regulates multiple neuronal functions through phosphorylation of serine and threonine residues of its target proteins [22]. It is abundant in the brain and participates in neurodevelopment, neurotransmitter synthesis and release, and cellular transport among other functions [23,24,25]. During dendrite formation, this kinase contributes to phosphorylation of microtubule-associated protein 2 (MAP2) [26,27,28].

CaMKII is activated by the main calcium-binding protein, calmodulin (CaM). This protein has a dumbbell shape connected by seven turn alpha helixes and two hydrophobic clefts that interact with its multiple targets [29]. CaMKII has three domains: the association domain located at the *C*-terminal region, the catalytic domain located at the N-terminal region, where the ATP binding site is positioned, and a regulatory domain placed in-between [22]. The Ca^2+^/CaM complex binds to the 293–310 amino acid sequence located in the regulatory domain, disrupting the autoinhibitory interaction between the regulatory and the catalytic domain [30]. After activation, this enzyme undergoes autophosphorylation at the threonine-286 residue, making its activity independent of Ca^2+^/CaM activation [31,32]. In addition, the kinase can be phosphorylated at serine-253, which allows its targeting into the cytoskeletal compartment [33]. Further, phosphorylation of thr-286 enhances its binding to MAP2 which plays a key role in dendrite formation [33,34].

Regulation of CaMKII activity depends on its relative concentration, as well as on the concentration of its activator Ca^2+^/CaM, among other factors. This enzyme can be enriched at specific subcellular compartments by translocation triggered by PKC activation [35]. Also, the relative concentration of CaM available to activate the kinase can be augmented by synthesis or targeting to specific subcellular sites. In this regard, MEL was shown to increase total cellular level and synthesis of CaM in epithelial cells after three days of incubation [36,37]. Also, it induces CaM subcellular redistribution and translocation from the cytosol to the membrane-cytoskeletal fraction in MDCK cells [36,38]. In addition, MEL can activate PKC downstream MT1/2 receptors, or directly in a non-receptor mediated pathway. Direct interactions between MEL and PKC have been described in neuroblastoma-cultured cells. Furthermore, the alpha isoform of PKC phosphorylates CaM in MDCK epithelial cells and induces changes in the subcellular distribution of this protein [39,40,41,42,43]. ERK1/2 was demonstrated to be involved in this signaling pathway downstream PKC [43]. This evidence suggested that PKC-mediated targeting of CaM to specific subcellular compartments can be implicated in the MEL regulation of CaM-dependent physiological processes. Thus, in this study we investigated whether MEL stimulates dendrite formation by CaMKII activation and increased CaM levels in organotypic cultures from adult rat hippocampus. To test this hypothesis, CaMKII was inhibited with the KN-62 compound and dendritogenesis was evaluated at three levels: dendrite formation, enlargement and complexity. Also, the relative levels of CaM were determined in homogenates and in the soluble and cytoskeletal fractions of hippocampal slices. In addition, participation of PKC and MEL receptors was explored by using bisindolylmaleimide, a PKC inhibitor, and luzindole, a MT1/2 antagonist. Besides, relative amount of autophosphorylated CaMKII, PKC and phospho-ERK1/2 was evaluated in hippocampal slices incubated with MEL. The results showed that both the CaMKII inhibitor and the PKC inhibitor abolished MEL effects on dendritogenesis. Also, phosphorylation of CaMKII, PKC and ERK1/2 was induced by MEL. Moreover, MEL increased the relative amount of CaM after a 6h-incubation in both total homogenates and in the soluble fraction of hippocampal slices. In addition, the MT1/2 antagonist partially inhibited MEL-induced dendritogenesis. Our results indicate that a physiological concentration of MEL prompts dendritogenesis through activation of CaMKII by increased levels of CaM in the soluble fraction. Also, data suggest participation of both MEL receptor-mediated and non-receptor-mediated mechanisms in dendritogenesis. Taken together, data strongly suggests that MEL could repair the loss of synaptic connectivity in neuropsychiatric patients, by augmenting dendrite availability in the hippocampus of the adult brain.

## 2. Results and Discussion

### 2.1. Ca^2+^/CaM Kinase II Participates in the Mechanisms by Which Melatonin Elicits Dendritogenesis

**Figure 1:**
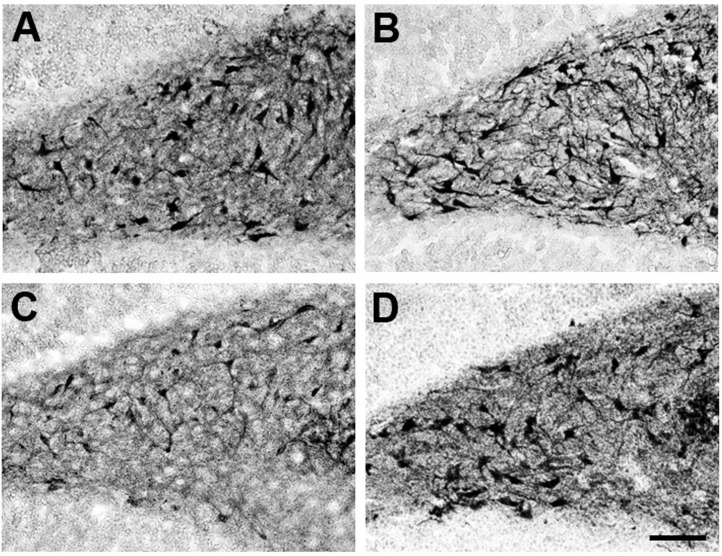
Effect of Ca^2+^/CaM-Kinase II on dendrite formation elicited by Melatonin. Participation of CaMKII on dendrite formation elicited by MEL was evaluated by specific inhibition of its activity with KN-62. Thus, rat brain hippocampus was cut in 400 µm slices and cultured in Neurobasal^®^ (GIBCO by Life Technologies, Grand Island, NY, USA) media for 7 days. Then they were incubated for 6 h with either the vehicle (**A**); 100 nM MEL (**B**); or pre-incubated with 10 µM KN-62 (**C**,**D**) followed by 6 h incubation with the vehicle (**C**) or 100 nM MEL (**D**). After the incubation time, slices were cut into 50 µM sections and immunostained for the specific marker of dendrites MAP2. Afterwards, slices were incubated with a secondary antibody coupled to biotin-avidin-peroxidase. Images were acquired with a digital camera coupled to a light microscope with the NIS-Elements software. Scale bar = 100 µm.

Previously it was demonstrated that MEL increases dendritogenesis in adult interneurons of the hilar region of the hippocampus and in new neurons formed in the dentate gyrus of rodents [17,18]. Also, it was shown that activation of CaMKII is involved in dendrite formation and maturation [23,44]. Thus, we tested whether the KN-62 compound, a specific inhibitor of CaMKII, suppresses the MEL effects on organotypic hippocampal cultures. As shown in Figure 1A, in presence of the vehicle, neurons of triangular shape with thin dendrites were evident. In presence of 100 nM MEL, neurons developed an intricate web of dendrites (Figure 1B). By contrast, hippocampal slices incubated with the CaMKII inhibitor and the vehicle, showed few and thin dendrites in the interneurons of the hilar zone (Figure 1C), while in hippocampal slices incubated with KN-62 and 100 nM MEL, only triangular and bipolar neurons with short and thin dendrites were evident and the complex dendrite network elicited by the indoleamine was not observed (Figure 1D). Since MEL-induced dendrite formation was abolished in the presence of the CaMKII inhibitor, data indicate that the enzyme participates in the mechanism by which MEL elicited dendritogenesis.

### 2.2. PKC Participates in Dendritogenesis Stimulated by Melatonin

**Figure 2:**
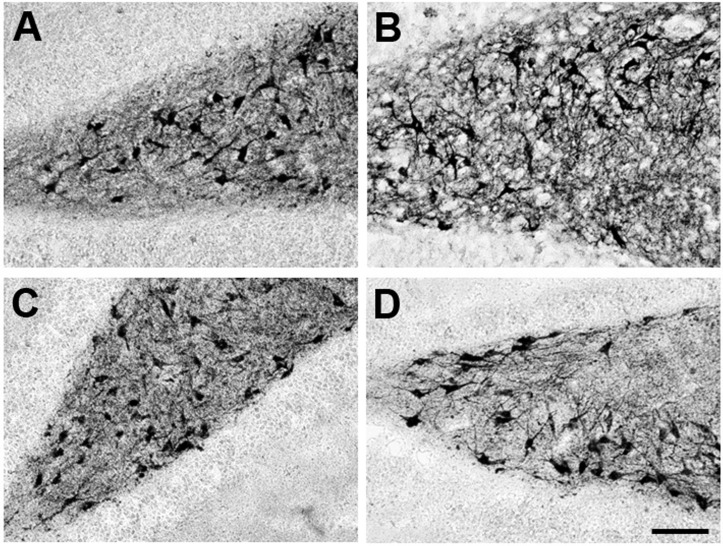
Effect of Protein Kinase C on dendrite formation elicited by Melatonin. Participation of PKC on dendrite formation elicited by MEL was evaluated by specific inhibition of its activity with bisindolylmaleimide. Thus, rat brain hippocampus was cut in 400 µm slices and cultured in Neurobasal^®^ media for 7 days. Then they were incubated for 6 h with either the vehicle (**A**); 100 nM MEL (**B**); or pre-incubated with 5 µM of bisindolylmaleimide (**C**,**D**) followed by 6 h incubation with the vehicle (**C**) or 100 nM MEL (**D**). After the incubation time, slices were cut into 50 µM sections and immunostained for the specific marker of dendrites MAP2. Afterwards, slices were incubated with a secondary antibody coupled to biotin-avidin-peroxidase. Images were acquired with a digital camera coupled to a light microscope with the NIS-Elements software. Scale bar = 100 µm.

The Ca^2+^/CaM complex is necessary for activation of CaMKII [45]. Also, PKC activated by MEL induced CaM phosphorylation which was followed by its subcellular redistribution in MDCK cells [43]. This suggests that PKC may contribute to CaM targeting to a specific subcellular compartment where it activates CaMKII, which in turn phosphorylates MAP2 to form dendrites. Thus, the next step to disclose the signaling pathway by which MEL activates CaMKII was to test the effect of bisindolylmaleimide, an inhibitor of the Ca^2+^-dependent PKC isoforms. As shown in Figure 2, in presence of the PKC inhibitor, no differences were evident in comparison with the vehicle-incubated organotypic cultures (Figure 2A,C). By contrast, in hippocampal slices incubated with bisindolylmaleimide and 100 nM MEL, a thinner dendrite network was observed in regard to the MEL-incubated hippocampal slices (Figure 2B,D). Data suggest that PKC is involved upstream of CaMKII in the signaling pathway by which MEL stimulates dendrite formation, and that its participation is crucial since no dendrites are formed when the enzyme is specifically inhibited.

### 2.3. Ca^2+^/CaM Kinase II and PKC Participate in Dendrite Formation, Enlargement and Complexity Elicited by Melatonin

**Figure 3:**
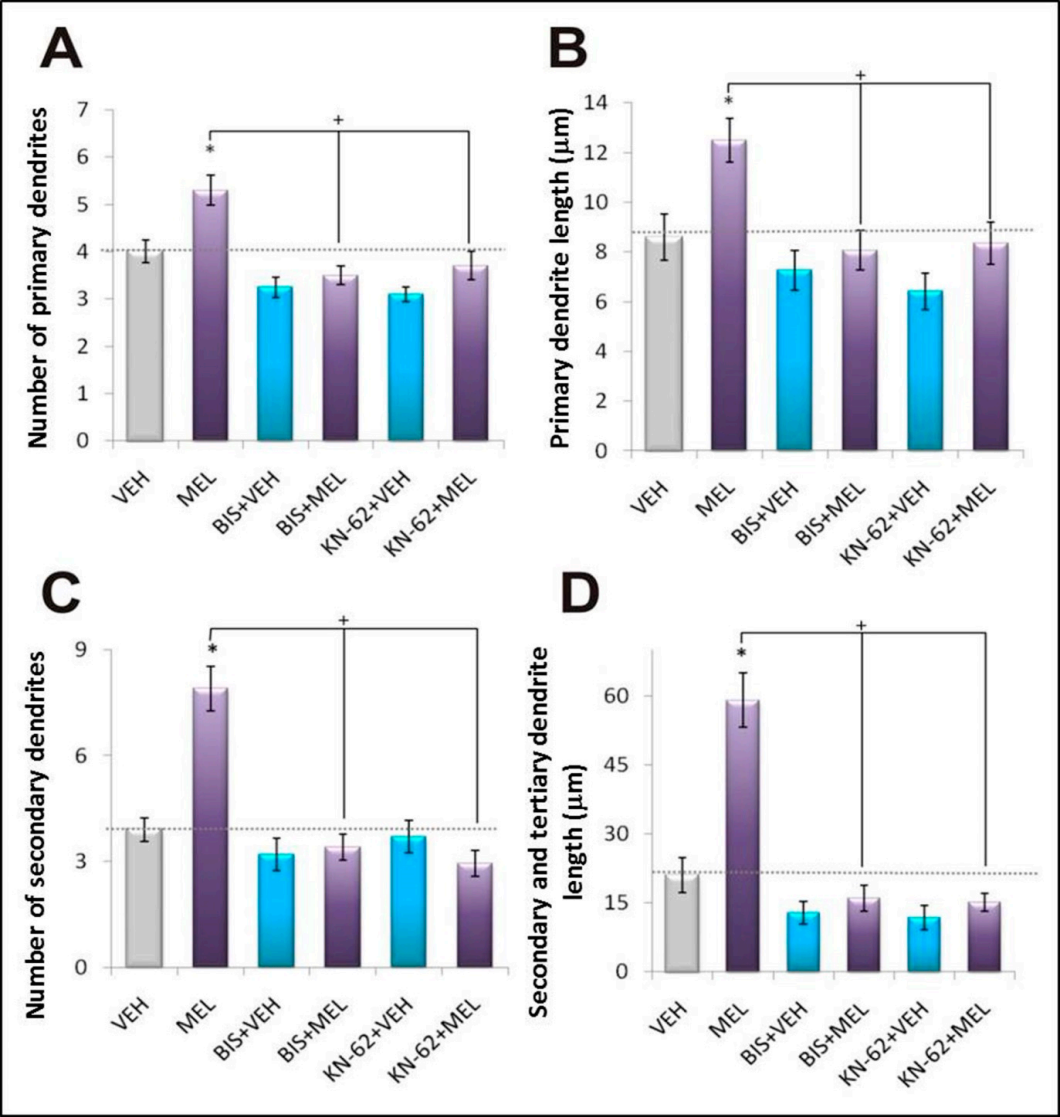
Morphometric analysis of dendritogenesis elicited by Melatonin: participation of Ca^2+^/CaM Kinase II and Protein Kinase C. The three stages of dendrite formation were evaluated by measuring the number and length of primary dendrites, as well as the number and length of secondary and tertiary dendrites. Hippocampal slices were incubated for 6 h with the following treatments: vehicle (VEH), 100 nM melatonin (MEL), pre- incubation of 15 min with 5 µM of the PKC inhibitor, bisindolylmaleimide (BIS) followed by the vehicle (BIS + VEH) or melatonin (BIS + MEL) for 6 h, or pre- incubation of 15 min with 10 µM of the CaMKII inhibitor, the KN-62 compound (KN-62) followed by the vehicle (KN-62 + VEH) or melatonin (KN-62 + MEL) for 6 h. After the incubation time, slices were cut into 50 µM sections and immunostained for the specific marker of dendrites MAP2. Slices were analyzed by the modified Sholl method. Results represent the mean ± SEM of one experiment of three done by quadruplicate. Asterisks show significant differences (*p* < 0.05). (**A**) Number of primary dendrites; (**B**) Primary dendrite length (µm); (**C**) Number of secondary dendrites; (**D**) Secondary and tertiary dendrite length (µm).

Dendrite development is a complicated process that involves three steps: formation, growth and complexity [46]. All three stages can be evaluated by measuring the number of primary dendrites, the length of primary dendrites, and the number and length of secondary dendrites, as an index of formation, outgrowth and complexity, respectively [18]. Thus, we determined those parameters and as shown in Figure 3, a decrease below basal levels was observed in the presence of either the CaMKII or PKC inhibitors. Also, no increase of dendrite formation, growth or complexity was observed in organotypic cultures incubated with 100 nM MEL and either of those inhibitors. Thus, morphometric analysis shows that all three stages of dendritogenesis were abolished by either the CaMKII or PKC inhibitors, supporting that both enzymes are in the signaling pathway by which MEL elicits dendritogenesis. Since KN-62 interacts with the Ca^2+^/CaM binding site of CaMKII impeding its activation, the results suggest that either increased CaM affinity or availability could be involved in MEL-induced dendritogenesis. In this regard, contradictory evidence has been reported about the ability of phosphorylated CaM to interact with its binding site in the regulatory domain of CaMKII. However, serine/threonine phosphorylation of CaM by casein kinase II increases its affinity for binding to CaMKII [47,48]. Thus, increased affinity by PKC-mediated phosphorylation on CaM serine/threonine residues or increased availability of CaM by augmented synthesis via PKC/cAMP response element binding protein or by targeting to specific subcellular sites can be involved in the mechanisms by which MEL stimulates dendritogenesis [22,29,47,49,50].

**Figure 4:**
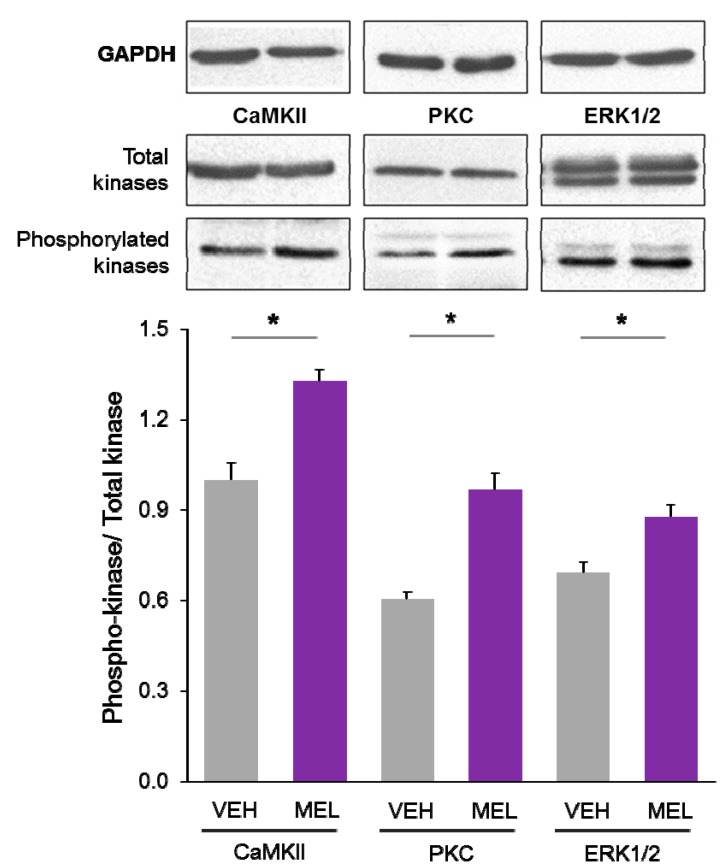
Relative content of phosphorylated Ca^2+^/CaM Kinase II, Protein Kinase C and Extracellular Signal-Regulated Kinase 1/2 in hippocampal slices incubated with Melatonin. Hippocampal slices were incubated for 3 h with either the vehicle (VEH) or 100 nM melatonin (MEL). CaMKII and Thr286-phospho-CaMKII (P-CaMKII), PKC and phospho-PKC (P-PKC) as well as ERK1/2 and phospho-ERK1/2 (P-ERK1/2) were determined by Western blot. Upper panels show immunodetection of GAPDH as load control. Representative fluorograms of total and phosphorylated enzymes revealed by ECL are shown below. Histograms correspond to densitometric analysis of bands depicted immediately above. Results are expressed as the mean ± SEM of four densitometric scannings obtained from two independent experiments. Asterisks indicate significant differences (*p* < 0.05).

Upon activation, PKC autophosphorylates in two different sites [51]. Similarly, Ca^2+^/CaM activation of CaMKII is followed by its intramolecular autophosphorylation in the threonine 286 residue [31,32] and ERK1/2 is phosphorylated after PKC activation in the hippocampus [52]. Therefore, to demonstrate that these enzymes are involved in dendritogenesis elicited by MEL, the relative levels of Thr286-phospho-CaMKII, phospho-PKC, and phospho-ERK1/2 were determined by Western blot. As shown in Figure 4, an increase of 32% in the phospho-kinase/total kinase ratio of CaMKII was evident in hippocampal slices incubated with 100 nM MEL. Also in this condition, the corresponding ratios of PKC and ERK1/2 were increased by 60% and 27% respectively. No increase in the amount of total CaMKII, PKC, ERK1/2 or GAPDH was evident (Figure 4 upper panels). CaMKII participation in the mechanism of action by which MEL increases dendritogenesis was demonstrated by blocking the enzyme activity with KN-62. However, because KN-62 may block the activity of various CaM kinases [32], autophosphorylation of CaMKII was determined to corroborate its activation by MEL treatment. Data clearly showed that the amount of autophosphorylated CaMKII was increased, supporting that MEL activates this enzyme. Similarly, an increase in both autophosphorylated PKC and phosphoERK1/2 was observed in hippocampal slices incubated with MEL, indicating that both enzymes are activated in the process of dendritogenesis elicited by this indoleamine.

### 2.4. Calmodulin Participation in the Mechanisms by Which Melatonin Elicits Dendritogenesis

**Figure 5:**
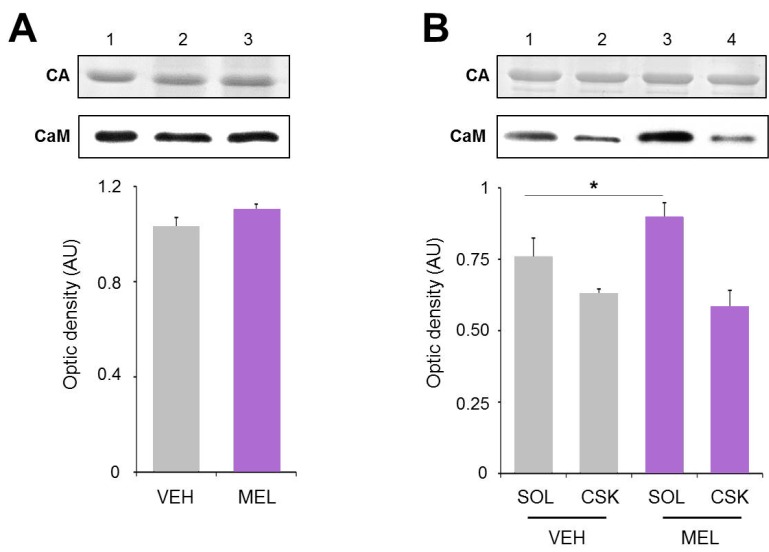
Calmodulin content in hippocampal slices treated with Melatonin. Hippocampal slices were incubated during 6 h with either the vehicle (VEH) or melatonin (MEL). Calmodulin (CaM) in the homogenates (**A**); and in the soluble (SOL) and cytoskeletal fractions (CSK) separated by centrifugation (**B**); was determined by Western blot. Upper panels show Carbonic Anhydrase (CA) used as external load control and stained with Coomassie blue. Representative fluorograms of CaM are shown immediately below. First lane of both gel and fluorogram from panel A was loaded with pure CA (5 µg) and CaM (1 µg), respectively. CaM was recognized with a specific CaM antibody and ECL. Histograms correspond to densitometric analysis of the bands shown in the upper panels. Results are the mean ± SEM of four densitometric scannings obtained from two independent experiments. Asterisk indicates significant differences (*p* < 0.05).

As mentioned before, one of the crucial conditions for CaMKII activation is the availability of Ca^2+^/CaM for binding and activating it. Therefore, as a first step to disclose the mechanism by which MEL stimulates the activity of CaMKII we determined CaM levels in total homogenates and in the soluble and cytoskeletal fractions of hippocampal slices incubated with either the vehicle or 100 nM MEL for 6 h. As shown in Figure 5A, a slight increase of 8% in total CaM content was observed after a 6 h-incubation with 100 nM MEL. No changes were observed in the carbonic anhydrase used as external load control (Figure 4, upper panel). Figure 5B shows the subcellular distribution of CaM in the soluble and cytoskeletal fractions of hippocampal slices. In the presence of MEL, a 43% increase in CaM was observed in the soluble fraction with a slight decrease in the cytoskeletal fraction. Data suggest that both an increase in synthesis and translocation might occur in presence of MEL, in agreement with previous observations made in epithelial MDCK cells where the indoleamine induced an increase in both parameters [36]. Moreover,* in vitro* assays have shown that MEL directly stimulates PKC alpha and phosphorylates CaM. In this regard, it is known that phosphorylation of proteins induces a conformational change that increases the affinity constant for specific protein binding and therefore, they are targeted to specific subcellular compartments [22]. Although our experimental strategy does not allow us to elucidate the mechanisms by which CaM is enriched in the soluble fraction, our data indicates that MEL might increase availability of CaM to activate CaMKII and therefore trigger dendrite formation.

### 2.5. MT1/2 Melatonin Receptors Participation in the Mechanisms by Which Melatonin Elicits Dendritogenesis

**Figure 6:**
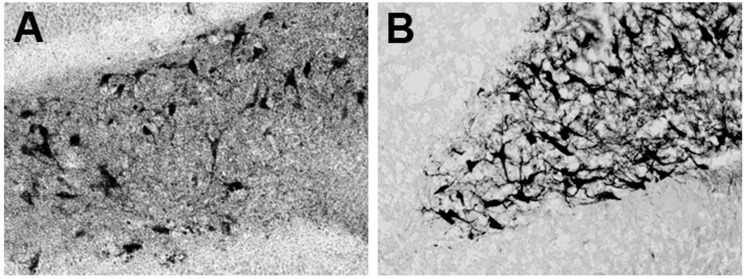

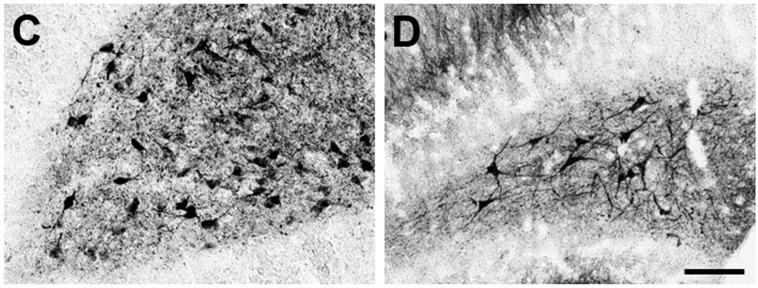
Participation of Melatonin receptors on dendrite formation elicited by Melatonin. To evaluate the involvement of MEL receptors, hippocampal organotypic cultures were incubated with the MT1/2 receptor antagonist, luzindole. Thus, rat brain hippocampus was cut in 400 µm slices and cultured in Neurobasal^®^ media for 7 days. Then they were incubated for 6 h with either the vehicle (**A**); 100 nM melatonin (**B**); or pre-incubated with 100 µM luzindole followed by 6 h incubation with the vehicle (**C**) or 100 nM melatonin (**D**). After the incubation time, slices were cut into 50 µM sections and immunostained for the specific marker of dendrites MAP2. Images were acquired with a camera coupled to a light microscope with the NIS-Elements software. Scale bar = 100 µm.

**Figure 7:**
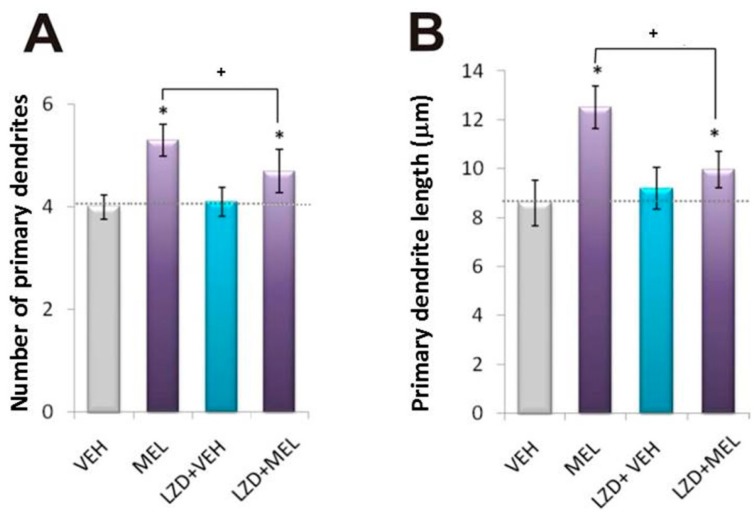

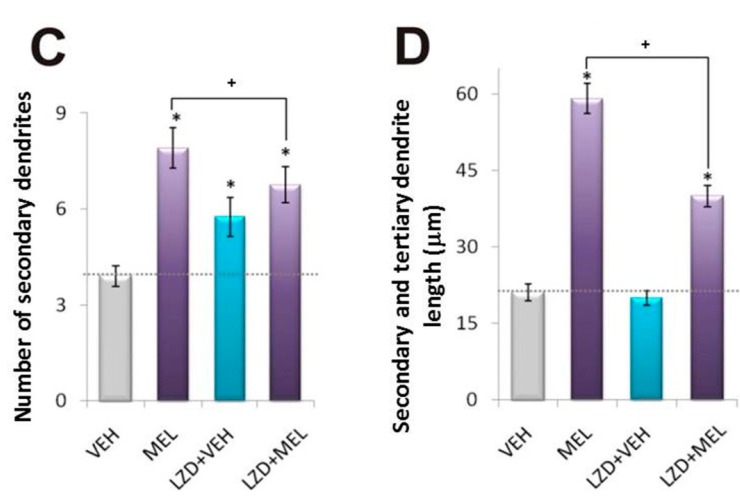
Morphometric analysis of dendrite formation elicited with Melatonin in presence of Luzindole. Hippocampal slices were incubated for 6 h with either the vehicle (VEH), 100 nM melatonin (MEL), or were pre-incubated with luzindole (LZD) for 15 min followed by 6 h incubation with the vehicle (LZD + VEH) or 100 nM melatonin (LZD + MEL). After the incubation time, slices were cut into 50 µM sections and immunostained for the specific marker of dendrites MAP2. Slices were analyzed by the modified Sholl method. Results represent the mean ± SEM of one experiment of three done by quadruplicate. Asterisks show significant differences (*p* < 0.05). (**A**) Number of primary dendrites; (**B**) Primary dendrite length (µm); (**C**) Number of secondary dendrites; (**D**) Secondary and tertiary dendrite length (µm).

Stimulation of MEL receptors (MT1/2) causes PKC activation that has been shown to be involved in physiological responses such as phase shifts in circadian rhythms [39]. Also it has been suggested that MEL directly stimulates PKC activity to modulate cytoskeletal assembly [41]. Thus, we studied the participation of MEL receptors upstream PKC in the MEL signaling pathway involved in dendrite formation. Therefore, the organotypic hippocampal slices were incubated with 100 µM luzindole, a MT1/2 competitive antagonist. Figure 6 shows images of hippocampal slices cultured with the MT1/2 receptor antagonist in presence of either the vehicle or MEL and stained with the MAP2 antibody. Luzindole had no effect on dendrite formation and similar dendrite staining pattern was obtained in the vehicle-incubated cultures with or without the MEL receptor antagonist (Figure 6A,C). By contrast, the organotypic cultures pre-incubated with luzindole followed by MEL showed fewer but enlarged dendrites in comparison with slices incubated only with MEL (Figure 6B,D). Histogram analysis of the effects of luzindole on the three stages of dendritogenesis is shown in Figure 7. Parameters of dendrite formation and growth were similar in the vehicle-incubated slices cultured with or without luzindole. (Figure 7A,B). However, the index of maturation determined as the number of secondary dendrites was stimulated by luzindole itself by 40% (Figure 7C). By contrast, in presence of MEL, luzindole blocked dendrite formation and enlargement by 50% and 65%, respectively (Figure 7A,B); while dendrite complexity evaluated through both the number and length of secondary dendrites was only decreased by nearly 30% (Figure 7C,D).

Since luzindole was tested at a concentration of 100 µM which is one order of magnitude higher to the K_i_ concentrations (nM) of MT1/2 receptors [53] and the hippocampus expresses both MT1 and MT2 receptors [54,55], our results suggest that both types of membrane receptors participate in dendrite formation triggered by MEL. However, since luzindole partially inhibited MEL-induced dendrite formation, enlargement and complexity (50%, 65% and 30%, respectively), this process must implicate other mechanisms and intracellular targets for MEL such as direct PKC activation that elicits microtubule assembly, a process which is also crucial for dendrite formation [56]. Interestingly, since luzindole stimulated the maturation of dendrites, it is possible that besides its antagonist action on MEL receptors, luzindole might act as an inverse agonist as previously described [57,58] to increase dendrite complexity.

## 3. Experimental Section

### 3.1. Hippocampal Organotypic Cultures

Male Wistar adult rats (52–56 days age, 200–250 g weight) were housed in polypropylene cages with food and water available *ad libitum* and exposed to a 12:12 h light-dark cycle. Animals were decapitated following all institutional and ethical regulations in conformity with international ethical standards. Brains were removed and placed in ice-cold artificial cerebrospinal fluid solution (aCSF) containing 124 mM NaCl, 5 mM KCl, 3.2 mM MgCl_2_, 25 mM NaHCO_3_, 10 mM Glucose, 0.09 mM CaCl_2_, 1.3 mM KH_2_PO_4_, pH 7.4, oxygenated with a mixture of 95% O_2_ and 5% CO_2_ [59]. They were cut into 400 µm coronal slices, from bregma −2.3 to −6.00 mm, using a Microm International vibratome (Thermo Scientific, Waltham, MA, USA), settings: 15–17 mm/s, 55 Hz and amplitude of 1.2. Hippocampal slices (4–6/insert) were placed onto membrane inserts of 0.4 µm pore size and 30 mm diameter (Millipore^®^, Billerica, MA, USA) [59,60,61] and cultured for one week in Neurobasal^®^ medium supplemented with 2% B27, 1% l-glutamine, and 1% penicillin-streptomycin antibiotics (Gibco Invitrogen™, Carlsbad, CA, USA).

### 3.2. Pharmacological Treatments

CaMKII participation in dendritogenesis elicited by MEL was studied by incubation of hippocampal slices with 10 µM KN-62 (1-[*N*,*O*-Bis(5-isoquinolinesulfonyl)-*N*-methyl-l-tyrosyl]-4-phenylpiperazine; Calbiochem^®^ EMD Chemicals, Gibbstown, NJ, USA), a specific inhibitor of the CaMKII activity [49]. PKC participation in dendrite formation was evaluated by using 5 µM bisindolylmaleimide GF109203X (Sigma-Aldrich^®^, St. Louis, MO, USA), an inhibitor of calcium-dependent PKC isoforms [62]. Organotypic cultures were pre-incubated for 15 min with either of the kinase inhibitors followed by 6h-incubation with either the vehicle or 100 nM MEL. The indoleamine was dissolved in 60 µL of ethanol and Neurobasal^®^ medium. Final ethanol concentration was 0.00004%. Kinase inhibitors were also dissolved in Neurobasal^®^ medium. Participation of MEL receptors in the mechanism by which the indoleamine induces dendritogenesis was approached by pre-incubation of slices during 15 min with 100 µM luzindole (Sigma-Aldrich^®^), a MT1/MT2 receptor antagonist [63], followed by 6 h incubation with either vehicle or 100 nM MEL.

### 3.3. Tissue Processing and Immunohistochemistry

To evaluate the architecture and number of dendrites formed in organotypic cultures subjected to the pharmacological treatments previously described, dendrites were stained with a specific antibody which recognizes the microtubule-associated protein 2 (MAP2) [26,64]. Therefore, cultured hippocampal slices were washed with PBS (138 mM NaCl, 2.68 mM KCl, 10.14 mM Na_2_HPO_4_, 1.76 mM KH_2_PO_4_, pH 7.4) and fixed with 4% paraformaldehyde for 1 day at 4 °C as described before [18]. Fixed slices were then incubated in 30% sucrose for three days, included in Neg-50™ medium (Richard-Allan Scientific™, Kalamazoo, MI, USA) and frozen for 30 min at −70 °C. Frozen slices were transferred to the Microm International cryostat (Thermo Scientific, Waltham, MA, USA) at −20 °C and 50 µm sections were cut and processed for immunohistochemistry as described [17,18,65]. Slices were incubated overnight at 4 °C with an anti-MAP2 antibody from Sigma-Aldrich^®^ Corporate diluted 1:200. Then, they were incubated with a secondary biotinylated anti-mouse antibody (Jackson Immunoresearch, West Grove, PA, USA) diluted 1:250. Slices were processed with Vectastain^®^ ABC Kit Elite Standard, and revealed with DAB/Ni Kit (Vector Laboratories, Burlingame, CA, USA) to obtain stained dendrites in black. Sections were clarified with Neoclear^®^ and mounted in Neomount^®^ medium (Merck, Darmstadt, Germany). Dendrite architecture of the hilar region of hippocampus was observed with a Nikon inverted microscope. Images were acquired with a Nikon camera and morphometric analysis performed with the NIS-Elements AR 3.0 software (Nikon, Melville, NY, USA).

### 3.4. Evaluation of Dendrite Formation by a Modified Sholl Method

**Figure 8:**
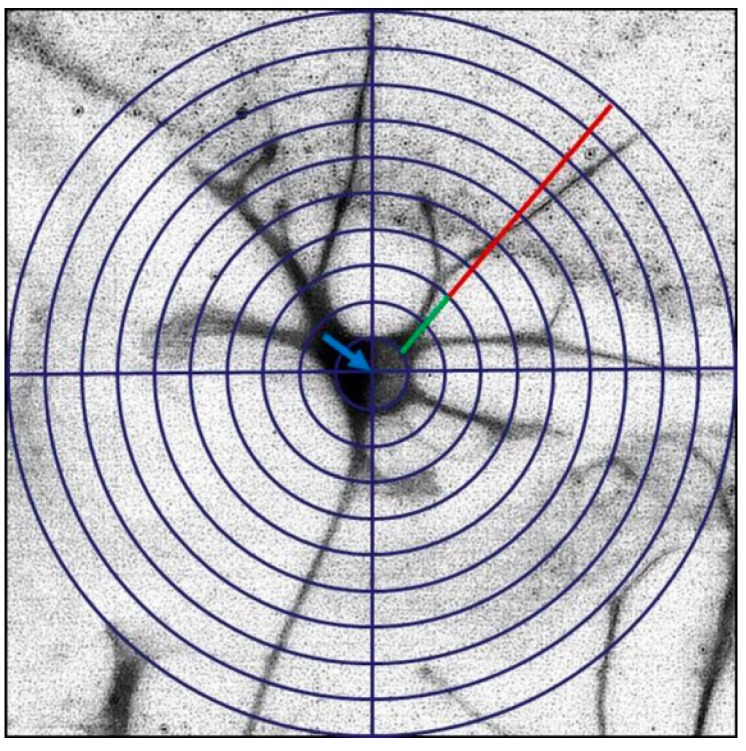
Schematic drawing of the modified Sholl method to determine the number and length of primary and secondary dendrites. A grid of concentric circles equidistantly drawn five micrometers apart was superimposed at the center of the neuronal soma (blue arrow). Dendrite formation was determined by counting the number of emerging dendrites from the soma. Primary dendrite length was measured from the soma up to the first intersection (green line). Complexity of dendrites was determined by counting the number of secondary dendrites as well as by measuring their length (red line).

Dendrite parameters were evaluated in MAP2-stained hippocampal slices visualized with an optic microscope, and analyzed with the Sholl modified method [18,66]. Briefly, after neuron identification in the hilar zone of the dentate gyrus, concentric and equidistant circles (step size of 5 µm) were used as a template and drawn superimposed around the soma (Figure 8). Hilar neurons with a soma diameter of 10 µm were selected for analysis. A neuronal projection of at least 5 µm-length that was immunostained for MAP2 was considered as a primary dendrite. Total dendrite length was determined by measuring the distance between the soma and the more distant tip. Primary dendrite length was determined by measuring the distance between the soma and the first intersection. Secondary and tertiary dendrite length was determined by subtraction of the primary or secondary dendrite length from the total dendrite length. The number and length of primary, secondary and tertiary dendrites were determined from 5 neurons per field by quadruplicate (20 hilar neurons per group).

### 3.5. Determination of Thr286-Phospho-CaMKII, Phospho-PKC and Phospho-ERK1/2 Relative Content

The relative amount of each kinase was assessed in 400 µm hippocampal brain slices obtained according to [67]. Hippocampal slices were placed in Millicel^®^ inserts and incubated with either the vehicle or 100 nM MEL during 3 h. Both the vehicle and MEL were prepared in Neurobasal^®^ medium supplemented with 1% B27, 200 mM glutamine and 1% penicillin-streptomycin as previously described. After incubation, slices were washed twice with PBS 0.01 M and then sonicated for 30 s, three times in an ice bath with RIPA buffer containing both protease and phosphatase inhibitors. Protein content from the homogenates was determined by Lowry’s method [68]. Proteins were separated in 10% polyacrylamide gels using the Laemmli buffer system [69]. Forty micrograms of total protein were loaded per lane. Proteins were transferred from the gel to 0.45 µm PVDF membrane by using the Towbin method [70]. Kinases were detected by Western blot. Membranes were blocked with 5% BSA-TBS (15 mM Tris, 137 mM NaCl, pH 7.5) for 1 h, and then incubated with either phospho-CaMKII (Thr286) antibody (CST^®^ #3361, Danvers, MA, USA), phospho-PKC (pan) (βII Ser660) antibody (CST^®^ #9371), or phospho-ERK1/2 (Thr202/Tyr204) antibody (CST^®^ #9106) diluted 1:1000 in 5% BSA-TBS-0.1% Tween 20, overnight, at 4 °C. After extensive washing, membranes were incubated with peroxidase-conjugated goat anti-rabbit IgG (Jackson Immunoresearch 111-035-003) or peroxidase-conjugated goat anti-mouse IgG (Jackson Immunoresearch 115-035-003) diluted 1:20,000, during 90 min at room temperature. Immunodetection of total CaMKII, PKC and ERK, as well as GAPDH as loading control were assessed using the following antibodies: anti-α-CaMKII (Invitrogen™ 13-7300), anti-PKCα (Santa Cruz Biotechnology^®^ sc-8393, Santa Cruz, CA, USA), anti-ERK1/2 (CST^®^ #9102) and anti-GAPDH (Millipore^®^ MAB374), respectively. After four washes, chemiluminiscent reaction with luminol peroxidase substrate reagent (Santa Cruz Biotechnology) was allowed for 1 min. Exposed films were revealed and fixed as previously described [71]. Fluorogram densitometric analysis was done using a GS800 densitometer and Quantity One Software (Bio-Rad, Hercules, CA, USA).

### 3.6. Determination of Calmodulin Relative Content

The relative amount of CaM was assessed in 400 µm hippocampal brain slices obtained according to [67]. Hippocampal slices were placed in Millicel^®^ inserts and incubated with either the vehicle or 100 nM MEL for 6 h. Both the vehicle and MEL were prepared in Neurobasal^®^ medium supplemented with 1% B27, 200 mM glutamine and 1% penicillin-streptomycin as previously described. After incubation, slices were washed twice with CSK buffer (10 mM PIPES, 2 mM EGTA, 100 mM KCl, 1 mM MgCl_2_, 20% glycerol) and then sonicated during 30 s for 3 times in an ice bath with CSK buffer containing 0.5% Triton X-100, 30 µg/mL aprotinin, 30 µg/mL leupeptin, 30 µg/mL pepstatin, 5 mM sodium pyrophosphate and 0.2 mM PMSF. Homogenates were then centrifuged twice at 13,500× *g* and soluble and cytoskeletal fractions were separated as described [72]. Cytoskeletal fraction was resuspended with the same volume of that of the soluble fraction. Protein content from both fractions was determined by Lowry’s method [68]. CaM was separated in 15% polyacrylamide gels using the Laemmli buffer system [69]. Sample loading buffer contained 5 mM EGTA [73]. Twenty micrograms of total protein and 5 µg of carbonic anhydrase, used as external load control, were loaded per lane. Proteins were transferred from the gel to 0.45 µm PVDF membrane by using the Towbin method modified with 2 mM CaCl_2_ according to McKeon and Lyman [70,74]. After transfer, membranes were incubated with 0.25% glutaraldehyde during 45 min for CaM fixation [75]. CaM was detected by Western blot. Membranes were blocked with 5% BSA-TBS for 1 h, and then incubated with mouse monoclonal anti-Calmodulin (SIGMA^®^ C7055) diluted 1:2000 in 1% BSA-TBS, overnight, at 4 °C. After extensive washing, membranes were incubated with biotin conjugated donkey anti-mouse IgG (Jackson Immunoresearch 715-065-151) diluted 1:12,000, during 90 min at room temperature. After three washes, avidin-peroxidase from Vector Laboratories (ABC kit) was added to membranes and incubated for 30 min. Chemiluminiscent reaction with luminol peroxidase substrate reagent (Santa Cruz Biotechnology) was allowed for 1 min. Exposed films were revealed and fixed as previously described [71]. Fluorogram analysis was done using GS800-densitometer and Quantity One software (Bio-Rad). Carbonic anhydrase was revealed by Coomasie blue staining of proteins in the polyacrylamide gels after CaM transfer. Densitometric analysis was performed as described before.

### 3.7. Statistical Analysis

Results are the mean ± standard error of the mean (SEM). Mean differences were analyzed by non-parametric test of Kruskal-Wallis ANOVA on Ranks followed by a Student-Newman-Keuls *post-hoc* test. Comparisons between two groups were done by Student’s *t*-test. Statistical analysis was performed using Sigma-Stat 3.1 software (San Jose, CA, USA). Differences were considered statistically significant at *p* < 0.05.

## 4. Conclusions

**Figure 9:**
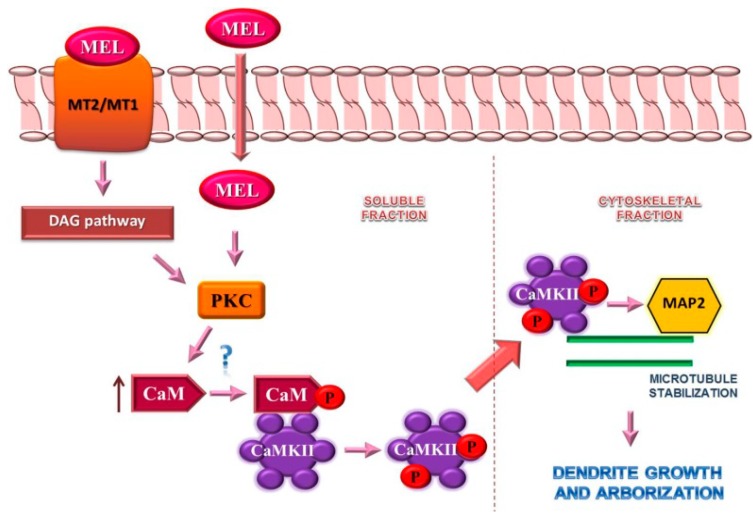
Schematic drawing of the signaling pathway by which Melatonin stimulates dendrite formation. Melatonin (MEL) binds to membrane receptors (MT2/MT1) to activate PKC through the diacylglycerol (DAG) pathway. Also, the indoleamine can diffuse through the plasmatic membrane to directly interact with protein kinase C (PKC). This enzyme may phosphorylate calmodulin (CaM), which can bind to Ca^2**+**^/CaM Kinase II (CaMKII) with higher affinity. CaM-activated CaMKII undergoes autophosphorylation which induces its targeting to the cytoskeletal compartment where it binds and phosphorylates MAP2 to constitute dendrites. Also, PKC may increase CaM levels in the soluble fraction by an enhanced biosynthesis or by targeting from the cytoskeletal to the soluble compartment.

In this paper we showed that both CaMKII and PKC participate in the mechanism by which MEL stimulates the three stages of dendritogenesis: dendrite formation, enlargement and complexity by two approaches: by pharmacological inhibition and by autophosphorylation. Our results also suggest that PKC acts upstream CaMKII and downstream to the MEL receptors and that ERK1/2 is involved in this signaling pathway. One important finding is that the CaM soluble pool is increased in hippocampal slices cultured with MEL indicating that CaM is available for CaMKII activation. Taken together, our data indicate that both receptor- and non-receptor-mediated signaling are concertedly involved in dendritogenesis triggered by MEL (Figure 9). More experiments are necessary for a more in-depth understanding of the mechanisms of CaM regulation exerted by MEL. Because dendrite formation is crucial for synaptogenesis, the results obtained in this study support that it is possible to stimulate neurodevelopment in the adult brain to reestablish the hippocampal synaptic connectivity lost in neuropsychiatric disorders.
